# Dielectric characterization and modelling of aqueous solutions involving sodium chloride and sucrose and application to the design of a bi-parameter RF-sensor

**DOI:** 10.1038/s41598-022-11355-w

**Published:** 2022-05-03

**Authors:** O. S. Bakam Nguenouho, A. Chevalier, B. Potelon, J. Benedicto, C. Quendo

**Affiliations:** Univ Brest, CNRS, Lab-STICC UMR 6285, 29200 Brest, France

**Keywords:** Electrical and electronic engineering, Sensors and biosensors, Design, synthesis and processing

## Abstract

This paper reports on dielectric properties of ternary mixtures involving sodium chloride (NaCl) and sucrose (C_12_H_22_O_11_) dissolved into water (H_2_O). Broadband electromagnetic characterizations of such mixtures at various concentrations were performed, evidencing a dual behavior made of conductive effects at low frequencies and dipolar relaxation at microwave frequencies. Conductive and dielectric properties resulting from these both effects were integrated into predictive models for variations of Cole–Cole model parameters. Based upon this modelling, an innovative microwave-based sensor able to retrieve concentrations of both sodium chloride and sucrose in ternary aqueous solutions was introduced, designed, realized and assessed. The proposed sensor shows an error lower than 5.5% for concentration ranges of 0 to 154 mmol/L for sodium chloride and 0 to 877 mmol/L for sucrose.

## Introduction

Nowadays, obtaining extensive, continuously-provided, accurate and real-time data is of key-importance for every system to work properly. For most systems, sensors are the data-generating elements and according to the increased demand for high resolution, good linearity and wide measurement ranges, expectations toward sensors are tremendously increasing^1,2^. These needs result in vivid research activities around the design of sensors, and particularly around sensors aimed to detect compound concentrations in liquids. Notably, this area is of key importance for applications such as food and drug industry^3–6^ or for biomedical application^7,8^. Tracking concentrations of specific compounds into solutions can rely on several physical principles such as chemical reactions, optical, electrical, or electromagnetic properties to cite a few. Chemical reactions are usually processed thanks to physico-enzymatic reactions, which necessitate destruction of the processed sample (usually picolitres of the solution) but feature very high resolutions^9–12^. Amongst non-destructive solutions, optical techniques have been intensively investigated, but they rely on the use of optically-transparent containers and/or solutions, which could be restricting^9,13,14^. Radio-frequency (RF) based sensors are recently attracting attention thanks to their invasivelessness and their ability to operate into optically-opaque media. They are focused on the tracking of various compounds spanning from temperature and humidity monitoring^15^, fluid characterisation^16,17^, oil and gas^18,19^, bio-liquids analysis^20^ to glucose sensing^21–23^.

Glucose sensing is a topic arousing high interest because of its numerous applications in chemical and biological systems. Controlling glucose level is a key-factor for the commercialization of drugs, food, etc. A lot of sensors based on RF-techniques to track glucose have been reported in the recent scientific literature, which have shown to be promising due to the penetration ability of the electromagnetic waves. Such techniques rely on the fact that sample concentration in a solution affects the dielectric properties of the solution. Any variation in those dielectric properties can be detected instantaneously, which opens the way for real-time sensing^24^. Some designs are based on broadband measurements such as coaxial probes, free space and waveguide^25–27^. This kind of measurement is usually large in size, and necessitates large samples to achieve reliable measurements (typically few centiliters), which could prevent them from being used in some harsh contexts.

Accordingly, techniques using low-volume samples are arousing attention, which lead to a lot of studies focusing on planar resonant techniques because of their ability to achieve measurements with relatively small samples^2,3,28^. In addition to the limited requested volume of liquid (typically few microliters), these planar resonant techniques draw attention thanks to their compactness, low cost and ease of manufacture. Furthermore, resonant RF-sensors usually exhibit greater sensitivities than broadband sensors. Several topologies can be found for resonant planar RF-sensors, from simple stub or LC resonator^29^ to more complex designs such as Split Ring Resonators (SRR)^30–32^ for example, which are quite convenient in sensing applications for combining the accuracy of the large structures while being miniaturized and highly sensitive. Although the sensing method of such sensors is usually based on the modification of the resonant frequency when in the presence of the sample to be characterized^3,15^, recent papers have shown that higher sensitivities can be reached thanks to the use of other indicators, such as the phase shift, magnitude of Scattering-Parameters or even Q-factors^33–37^.

Majority of those papers and the studies found in the literature are focused on the design of sensors able to track concentration of one given compound dissolved into a solution or a complex media. Some of them investigate or give clues about the cross-sensitivities of the tracked parameter with respect to other parameters such as temperature for instance. However, to our knowledge, very few papers report on the design of RF sensors dedicated to the tracking of several separate parameters with one single device in spite of the major interest it can provide for analyzing complex media such as those found in many applications.

A recently-published study focusing on sodium chloride and glucose sensing has been proposed^38^, it proposes to track concentrations of ternary mixtures through the visualization of microwave near-field distribution achieved thanks to thermo-elastic optical indication. As this work relies on a hybrid microwave/optical technique, it requires a quite complex set up and suffers from the optical methods classical drawbacks.

In the present paper, we propose to investigate the design of a single RF-based sensor purposed to determine the respective concentrations of two distinct compounds dissolved into an aqueous solution. Novelty of this paper is to give a proof of feasibility of such a dual-parameter sensor, its sensing abilities being exclusively based on electrical measurements. The proposed compounds to be tracked are sodium chloride and sucrose, which, as a first approximation, modify ionic behavior and dipolar relaxation, respectively. These intrinsic differences, together with the fact that these substances are widely used and found in plenty of applications have driven our choices. Indeed, sodium chloride and sucrose play a fundamental role in many chemical processes in a wide variety of domains.

Sodium chloride is an ionic compound, its molecular formula is NaCl. In its edible form of table salt, it is commonly used as a condiment and food preservative, it is largely found in nature as it is mainly responsible for the salinity of the seas. Large quantities of sodium chloride are used in many industrial processes, and it is a major source of sodium and chlorine compounds used as feedstocks for further chemical syntheses.

Sucrose is commonly known as table sugar, and is obtained from sugar cane or sugar beets, therefore, it is found in abundance in nature. Sucrose is a disaccharide formed from the monosaccharide glucose associated with a monosaccharide fructose. The molecular formula of sucrose is C_12_H_22_O_11_^39,40^.

Accurate detection and quantification of sucrose and sodium chloride concentrations in water with high sensitivity, selectivity and accuracy is thus of great interest. Notwithstanding the primary aim of this work was to demonstrate the feasibility of an RF-based sensor to track both sodium chloride and sucrose in aqueous solutions, concentration ranges consistent with those found in the food and beverage industry^41–43^ were targeted (0–9 g/L, which stands for 0–154 mmol/L for sodium chloride, and 0–300 g/L, which stands for 0–877 mmol/L for sucrose) to highlight the applicative interest of this study.

Although the Electro-Magnetic (EM) properties of sodium chloride and sucrose are different, with ionic conductivity on one hand and exhibiting a dipolar relaxation on the other hand, these species interact when they are mixed together into a solution, especially through the modification of the viscosity of the solution. It is thus paramount to study these physical interactions and their impact over their respective dielectric signatures. To reach this objective, direct electromagnetic characterizations were performed. They allowed the obtention of a thoroughful EM models for ternary mixes involving water, sodium chloride and sucrose.

EM Characterization, analysis and model of binary and ternary mixes are presented in the next section of this paper.

Then, based on these EM models, and their specific features, a sensor was designed and realized, which is presented in “Sensor design” section. “Experimental assessment of the sensor” section is devoted to the measurements and assessment of the fabricated sensor together with discussion of the obtained results. Finally, conclusions and future scopes are proposed in the last section.

### Dielectric characterization and modelling of aqueous solutions

The polarization undergone by a material when an electrical field is applied is referred as dielectric permittivity; it is generally given as a complex value:

1 $$ \varepsilon^{*} \left( \omega \right) = \varepsilon^{\prime}\left( \omega \right) - j\varepsilon^{\prime\prime}\left( \omega \right), $$

where $\omega$ is the angular frequency and *j* the imaginary unit.

The real part ${\varepsilon }^{{\prime}}$ of the dielectric permittivity is a measure for the polarizability of a material and the imaginary part $\varepsilon^{\prime\prime}$, is a measure for dielectric losses^25,44,45^. In aqueous solutions, the polarizability originates from rotation of the dipole moment of water molecule and the large value of *ε'* comes from cooperative motions due to intermolecular hydrogen bonds^46,47^. The dielectric losses *ε"* mainly arise from the dipole relaxation, which occurs at microwave frequencies. Moreover, for aqueous solutions with dissolved salts, additional conductive losses arising from ion mobility occur at low frequencies. The most commonly used model to describe the complex permittivity of aqueous solutions is the Cole–Cole model, a semi-empirical modification of the Debye dipolar relaxation model, in which a term related to the ionic conductivity is added to account the ions mobility^48^.

2 $${\varepsilon }^{*}\left(\omega \right)={\varepsilon }_{\infty }+\frac{{\varepsilon }_{stat}-{\varepsilon }_{\infty }}{1+{\left(j\omega \tau \right)}^{1-\alpha }}+\frac{{\sigma }_{s}}{j\omega {\varepsilon }_{0}},$$

where ${\varepsilon }_{\infty }$ and ${\varepsilon }_{stat}$ are the limits of the relative dielectric permittivity at low and high frequencies, respectively, $\omega$ is the angular frequency, $\tau$ is the relaxation time, ${\sigma }_{s}$ is the static ionic conductivity, ${\varepsilon }_{0}$ is the free space permittivity $(8.854\times {10}^{-12}\,\text{F}/\text{m})$ and $\alpha$ is a dimensionless exponent parameter taking into account dispersion in relaxation time. For De-Ionized (DI) water at room temperature (23 °C) the commonly accepted values of Cole–Cole parameters^49^ are: ε_∞_ = 5.1, ε_stat_ = 79.2, τ = 8.72 ps, $\alpha$= 0 and σ_S_ = 0 mS/cm. Most aqueous solutions with dissolved solutes can thus be modelled using Eq. (2), whose parameters depend on solute concentrations and other quantities such as temperature or pressure.In this work, we have chosen to study two common solutes that influence the behavior of the permittivity in different ways. Firstly, sodium chloride was used, it adds dissolved ions and mainly changes the conductive behavior of the solution. Secondly, sucrose was used, it increases the viscosity and mainly modifies the dipolar behavior of the solution.

In next parts, we propose to extract the concentration dependency of parameters of Eq. (2) at room temperature and pressure; firstly for sodium chloride solutions, then for sucrose solutions and finally for mixtures of both sodium chloride and sucrose.

**Figure 1:**
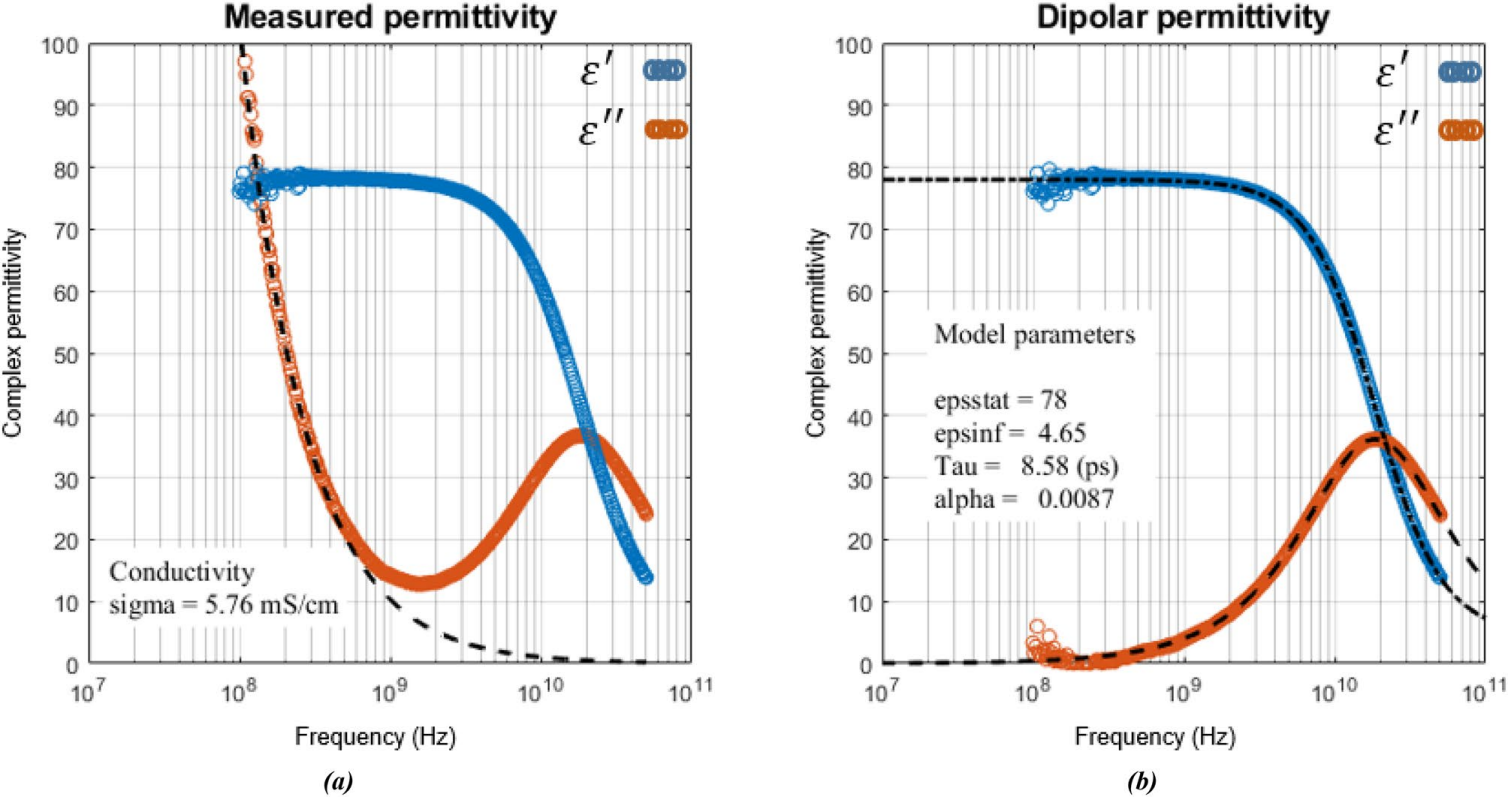
Complex permittivity of an aqueous solution: measured (o) and theoretical (–) values. (**a**) Determination of ionic conductivity using curve fitting of the imaginary permittivity in low frequency. (**b**) Determination of Cole–Cole parameters using fitting of the dipolar contribution.

To do this, broadband permittivity measurements were performed on aqueous solutions using the open-ended coaxial probe method (Keysight^®^ N1501A-104 probe and Agilent^®^ E8364 PNA-L Network Analyzer)^27^ in the [100 MHz–50 GHz] frequency range at room temperature (23 °C). A Matlab^®^ curve fitting code was developed to extract values of Cole–Cole model parameters that best fit the measured complex permittivity. Taking advantage that the variations of the imaginary part at low frequency are important but mostly depend on the conductive effects we chose a two-step fitting procedure to reduce optimization uncertainties and improve the accuracy of optimized parameters ε_∞_, ε_s_, τ, α and σ_s_. First, the conductivity σ_s_ is determined from fitting the imaginary part of the permittivity at low frequency (Fig. 1a). Then the conductive behavior is subtracted from the measurement according to Eq. (2) to keep only the dipolar behavior. Finally, the model parameters ε_∞_, ε_s_, τ and α are determined from fitting both imaginary and real parts of the dipolar permittivity (Fig. 1b). In addition, measurements of static ionic conductivity were performed using a SevenExcellence™ EC meter from Metter Toledo to validate the method. Agreement between static measured conductivity values and those determined from dynamic permittivity is better than 2%. Figure 1 shows an example of the two-step fitting procedure to extract the model parameters from permittivity measurement of a salt water solution.

Note that in Eq. (2), ε_∞_ is a mathematical parameter that represents the dielectric permittivity at high frequencies but does not correspond to the actual physical value of permittivity in the infrared frequency range^49,50^. The refractive index $n=\sqrt{{\varepsilon }_{\infty }}$ calculated from the infinite permittivity of the Cole–Cole model (ε_∞_ = 5.1) is different from the 1.33 measured by optical techniques. Other relaxation and resonance processes appear at high frequencies that are not taken into account in the Cole–Cole model^49^. The infinite permittivity in the Cole–Cole model only describes the rate decrease in permittivity at microwave frequencies. Although the value of ε_∞_ is sometimes fixed to the water one in the literature^51^, we decided to use it as an optimization parameter to best fit the experimental curves in our frequency range of interest without relating its variation to physical phenomena.

#### Sodium chloride aqueous solution

Due to its large polarity, water is an excellent solvent that is able to dissociate salts by separating the cations and anions and forming new interactions between the water and ions. The dielectric properties of sodium chloride aqueous solutions have been intensively studied in the literature^51–53^. The sodium chloride dissolves to sodium cation and chloride anion, thus providing an ionic character and helping to make the solution conductive.

In the presence of an electric field, ions move according to their mobility, which strongly depends on the viscosity^51^ of the solution. Indeed, ionic conductivity is proportional to the concentration of sodium chloride in the solution and inversely proportional to the viscosity^54–56^. It is worthwhile to note that the temperature influences the viscosity, this is highlighted in various research works^28,57,58^.

In addition to ions that generate a conductive effect, the sodium chloride aqueous solutions also exhibit a dipolar relaxation alike the DI water one. This dipolar relaxation being related to the viscosity of the solution, it is also influenced by temperature and solute concentrations. To evidence these effects on EM properties, permittivity characterization of sodium chloride aqueous solutions was performed.

**Figure 2:**
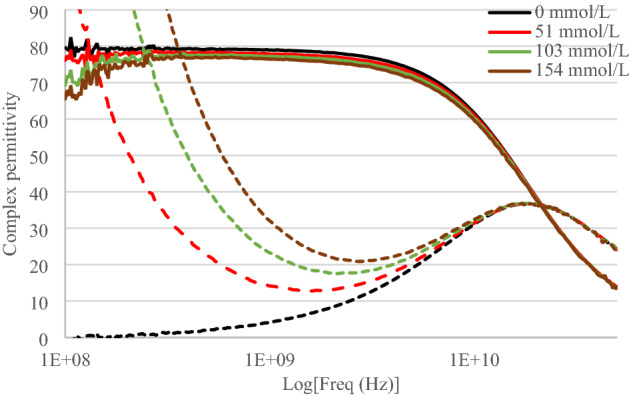
Real part (solid line) and imaginary part (dashed line) of the complex permittivity as a function of frequency (logarithmic scale) for various sodium chloride concentrations.

Solutions were made by diluting sodium chloride from Merck^®^ (6404) into DI water. Sodium chloride was weighted thanks to a precision balance from Kern Tab^®^. Obtained concentrations of sodium chloride in the solution were respectively 51 mmol/L, 103 mmol/L and 154 mmol/L. The measured complex permittivity of sodium chloride aqueous solutions is shown in Fig. 2.

For non-zero sodium chloride concentrations, the imaginary part of the permittivity exhibits two regions of losses. First at low frequencies (up to 1 GHz approximately), we observe a decrease in the imaginary part inversely proportional to the frequency and proportional to the sodium chloride concentration corresponding to the ionic conductive losses. Then at high frequencies, we observe the losses of dipolar relaxation of water molecules with a maximum of the imaginary part occurring around 18 GHz whatever the concentration.

The real part of the permittivity slightly decreases as sodium chloride concentration increases. For high concentrations, measurement uncertainties appear at low frequency due to the electrode polarization effects^59^. So the first frequency points of measured permittivity were ignored for the curve fitting of high concentrations.

From these measurements, we observe, as expected, that sodium chloride clearly modifies the ionic behavior of the permittivity and hardly modifies the dipolar behavior. The extracted parameters of the model are given in Table 1 for each concentration.

**Table 1:** Extracted Cole–Cole model parameters for sodium chloride/water solutions.

C_NaCl_ (mmol/L)	${\varepsilon }_{\infty }$	${\varepsilon }_{s}$	$\tau\, (\text{ps})$	α	$\sigma\, \left(\text{mS}/\text{cm}\right)$
0	4.60	79.20	8.71	0.011	0
51	4.00	78.20	8.52	0.016	5.76
103	3.20	77.50	8.41	0.024	10.92
154	2.85	76.90	8.36	0.030	15.99

From this table, one can note that the static permittivity ε_stat_ is slowly decreasing as the sodium chloride concentration increases. This is the consequence of, first, the lower concentrations of water when sodium chloride concentration increases and, second, of the ion–solvent interaction which leads to non-rotational bonding of water molecule in the vicinity of ions.

The relaxation time τ slightly decreases as sodium chloride concentration increases. This behavior is quite unexpected because increasing the solute concentration generally leads to increase the viscosity and thus to decrease the relaxation time. However, in sodium chloride solutions, viscosity increases very slightly with concentration^60^. Moreover, two antagonist contributions are present, a relaxation time of water surrounding cation (τ+) higher than those of DI water and a lower relaxation time of water surrounding anion (τ−)^61^. The summation leads to a slightly decreasing mean relaxation time for sodium chloride concentrations below 1 mol/L^51^.

The ionic conductivity ${\sigma }_{s}$ considerably increases as the sodium chloride concentration increases. As the viscosity of sodium chloride solutions shows very little changes, the conductivity behaviour is mainly driven by the amount of ions added to the DI water.

The parameter α slightly increases as the sodium chloride concentration increases. The presence of solutes in solution leads to a broadening of the relaxation losses that is well described by dispersion in relaxation time.

The parameter ${\varepsilon }_{\infty }$ decreases slightly as the concentration increases but since it acts as a mathematical optimization parameter, no physical phenomena was associated to its variation.

The parameters extracted from our measurements on sodium chloride aqueous solutions (Table 1) were compared to those found in the literature^51^*.* The values we obtained and their variations with solute concentration are in agreement with previously-published models.

To summarize, sodium chloride diluted in water mainly provides a conductive character due to dissolved ions, and only slightly affects the dipolar character due to water molecules. Note that the concentration of sodium chloride can be determined by knowing only one parameter such as the static ionic conductivity of the solution.

Now let us investigate the dilution of a non-ionic solute in water, and its effects on the Cole–Cole model parameters.

#### Sucrose/water mixtures

Sucrose molecule was chosen because it greatly affects the viscosity of aqueous solutions and does not exhibit ionic character. When diluted into water, the dipolar character of the solution mainly comes from the water molecules, but it is greatly affected by the viscosity modifications brought by sucrose. There is a large volume of published studies describing the viscosity properties of sucrose aqueous solutions^58,62^. However, there are relatively few studies in the area of permittivity modelling of sucrose solutions. A work^63^ studied the permittivity dependency on solute concentration, but this study was limited to static case only. Another published work^64^ proposes the study of the complex permittivity of carbohydrate aqueous solutions, but only for one concentration (33% w/w = 1.1 mol/L) at a low temperature of 5 °C. So, to evidence effects of sucrose on EM properties, permittivity characterization of sucrose aqueous solutions was performed.

Solutions were made from pure sucrose dissolved into DI water. Sucrose was weighted thanks to a Precisa XB1200C precision balance. Obtained concentrations of sucrose in the solutions were respectively 293 mmol/L, 585 mmol/L and 879 mmol/L.

**Figure 3:**
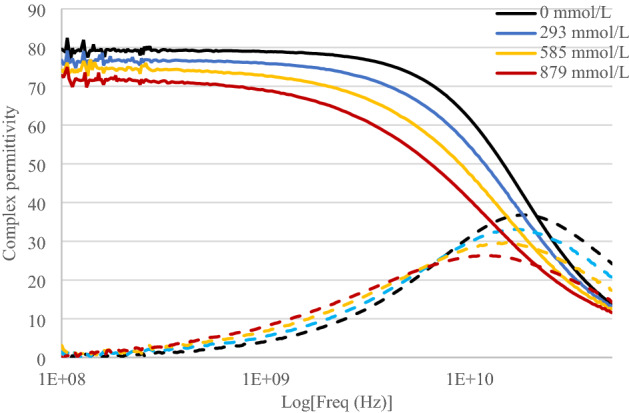
Complex permittivity (real part (line) and imaginary part (dash line)) as a function of frequency (logarithmic scale) for various sucrose concentrations.

Figure 3 shows the permittivity measurement of sucrose aqueous solutions at various concentrations over a frequency range starting at 100 MHz and up to 50 GHz.

As expected, no conductivity effects on these measurements of permittivity can be seen. The imaginary part of the permittivity exhibits only one region of losses at high frequencies corresponding to the relaxation process. However, the shift towards low frequencies of the maximum of the imaginary part shows the effect of viscosity brought by sucrose. Moreover, the maximum values of both real and imaginary parts of the permittivity significantly decrease as sucrose concentration increases. Table 2 summarizes the values of model parameters extracted from measurements at each concentration of sucrose.

**Table 2:** Extracted Cole–Cole model parameters for sucrose/water solutions.

${{C}_{{\text{C}}_{12}{\text{H}}_{22}{\text{O}}_{11}}}$ $(\text{mmol}/\text{L})$	${\varepsilon }_{\infty }$	${\varepsilon }_{stat}$	$\tau\, (\text{ps})$	α	$\sigma$ $\left(\text{mS}/\text{cm}\right)$
0	4.60	79.20	8.71	0.011	0
293	2.14	76.90	9.55	0.075	0
585	1.43	74.80	11.2	0.130	0
879	1.55	72.30	13.6	0.180	0

The static permittivity ε_stat_ significantly decreases as the sucrose concentration increases. As for sodium chloride, this is a consequence of the lower concentrations of water molecules when sucrose concentration increases. Moreover, the sucrose molecule is large and its first hydration shell contains over 37 water molecules^65^ leading to much more non-rotational bonds than for sodium chloride. That is why the decrease of the static permittivity is important for sucrose aqueous solutions. Measured values of static permittivity are in agreement with those published in the literature^63^.

The relaxation time $\tau$ is greatly impacted by sucrose concentration because it strongly affects viscosity^58^, reducing water molecules mobility and thus their ability to relax quickly when the dynamic electric field is applied. Thus, the relaxation time significantly increases as the sucrose concentration increases.

The static conductivity ${\sigma }_{s}$ is null as sucrose does not introduce any ionic contribution in aqueous solutions.

The parameter α increases significantly as the sucrose concentration increases. The widening of the imaginary part of the permittivity spectra is greater than for sodium chloride solutions because the viscosity changes brought by sucrose are greater.

As for sodium chloride solution, the parameter ${\varepsilon }_{\infty }$ decreases as sucrose concentrations increase.

We have shown that, as expected, sucrose significantly modifies the dipolar behavior and does not modify the conductive behavior of the permittivity. Note that the concentration of a sucrose aqueous solution can be determined by knowing only one parameter such as the static permittivity of the solution.

Now that effects over EM characteristics of sodium chloride aqueous solutions on one hand and sucrose aqueous solutions on the other hand, have been described, we propose in the next section to investigate the behavior of ternary solutions involving both sodium chloride and sucrose diluted into water.

#### Ternary mixtures involving water, sodium chloride and sucrose

In ternary solutions made from sodium chloride, sucrose, and water, several physical phenomena occur and interactions between them appear. Especially, the viscosity brought by sucrose significantly affects the ionic conductivity brought by sodium chloride. Although different studies exist in the literature regarding the viscosity or conductivity of such ternary solutions, none deals with the change of permittivity according to interactions between sucrose and sodium chloride in aqueous solutions.

To identify and quantify these interactions, broadband (100 MHz–50 GHz) permittivity measurements of ternary mixtures made up from sodium chloride, sucrose and water were performed at room temperature (23 °C). Solutions were prepared using the same compounds, the same procedure and the same equipment as in the previous sections. Targeted solutes concentrations are close to 0 mmol/L, 50 mmol/L, 100 mmol/L and 150 mmol/L for the sodium chloride and 0 mmol/L, 290 mmol/L, 580 mmol/L and 870 mmol/L for the sucrose concentrations.

To better identify the solutions, one letter is attributed to each sodium chloride concentration whereas one number is associated to each sucrose concentration. Thereby, A0 stands for DI-water, samples labelled B0, C0 and D0 are binary mixtures made from sodium chloride and water, as studied in “Sodium chloride aqueous solution” section and samples labelled A1, A2, A3 are binary mixtures made from sucrose and water, alike those presented in “Sucrose/water mixtures” section. All other labels with letter different than A and number different than 0 are ternary mixtures.

**Figure 4:**
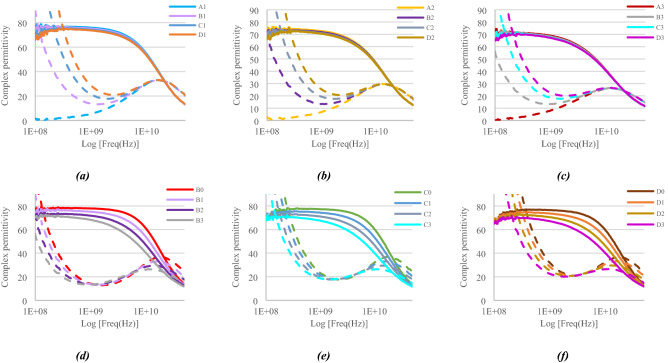
Complex permittivity of samples for given sucrose concentrations (**a–c**) and for given sodium chloride concentration (**d–f**) as a function of frequency (logarithmic scale).

Figure 4 shows the variation of measured complex permittivity of ternary mixtures over the frequency range 100 MHz–50 GHz.

As in the previous sections, we use the curve fitting method to extract the model parameters from these measurements. Table 3 shows the resulting parameters.

**Table 3:** Extracted Cole–Cole model parameters for ternary mixtures.

Solution samples	C_NaCl_ (mmol/L)	${{C}_{{{\text{C}}_{12}{\text{H}}_{22}{\text{O}}_{11}}}}$ (mmol/L)	${\varepsilon }_{\infty }$	${\varepsilon }_{stat}$	$\tau \,(\text{ps})$	α	σ_s_ (mS/cm)
A0	0	0	4.60	79.20	8.71	0.011	0
B0	51	0	4.00	78.20	8.52	0.016	5.76
C0	103	0	3.20	77.50	8.41	0.024	10.92
D0	154	0	2.85	76.90	8.36	0.030	15.99
A1	0	293	2.14	76.90	9.55	0.075	0
B1	52	294	1.88	76.30	9.56	0.084	4.77
C1	104	293	1.30	75.40	9.45	0.085	9.11
D1	154	292	0.70	74.80	9.15	0.096	13.22
A2	0	585	1.43	74.80	11.2	0.130	0
B2	51	585	1.09	73.90	11.0	0.140	3.80
C2	103	586	0.38	73.40	10.8	0.150	7.26
D2	154	585	0.10	73.00	10.8	0.150	10.53
A3	0	879	1.55	72.30	13.6	0.180	0
B3	51	877	0.98	72.00	13.5	0.190	2.92
C3	106	877	0.30	71.50	13.2	0.200	5.82
D3	154	877	0.10	71.10	13.0	0.200	8.12

We observe that the static permittivity ε_stat_ decreases when either the sodium chloride or the sucrose concentrations increase, because of the same reasons as for binary mixtures. In ternary mixtures, the decrease of ε_stat_ is mainly due to the concentration of sucrose but is emphasized by the sodium chloride concentration.

As for binary mixtures, the relaxation time τ of ternary mixtures is strongly impacted by sucrose due to its strong impact on viscosity. Thus, the relaxation time increases significantly as the sucrose concentration increases but is less influenced by sodium chloride. For a fixed sucrose concentration, the relaxation time decreases slightly as the sodium chloride concentration increases for the same reasons as those given in “Sodium chloride aqueous solution” section.

Viscosity effects brought by sucrose are greatly noticeable on the ionic conductivity. For example, by considering a sodium chloride concentration set at 154 mmol/L, the conductivity σ_S_ decreases from 15.99 to 8.12 mS/cm as the sucrose concentration increases from 0 to 877 mmol/L respectively. The conductivity ${\sigma }_{s}$ of ternary solutions is mainly related to the amount of solvated ions: the greater the concentration of sodium chloride the greater the conductivity. However, viscosity reduces the ion mobility: the higher the sucrose concentration, the lower the conductivity.

As in binary mixture, the greater the concentration of sucrose in ternary mixture, the greater the $\alpha$ parameter. In addition, $\alpha$ slightly increases as the sodium chloride concentration increases. Thus, the higher the solute concentration in ternary mixture, the wider the loss curves.

Concerning parameter *ε*_∞_, the greater the concentration of sucrose, the lower the parameter. In addition, *ε*_∞_, decreases slightly as the sodium chloride concentration increases.

**Figure 5:**
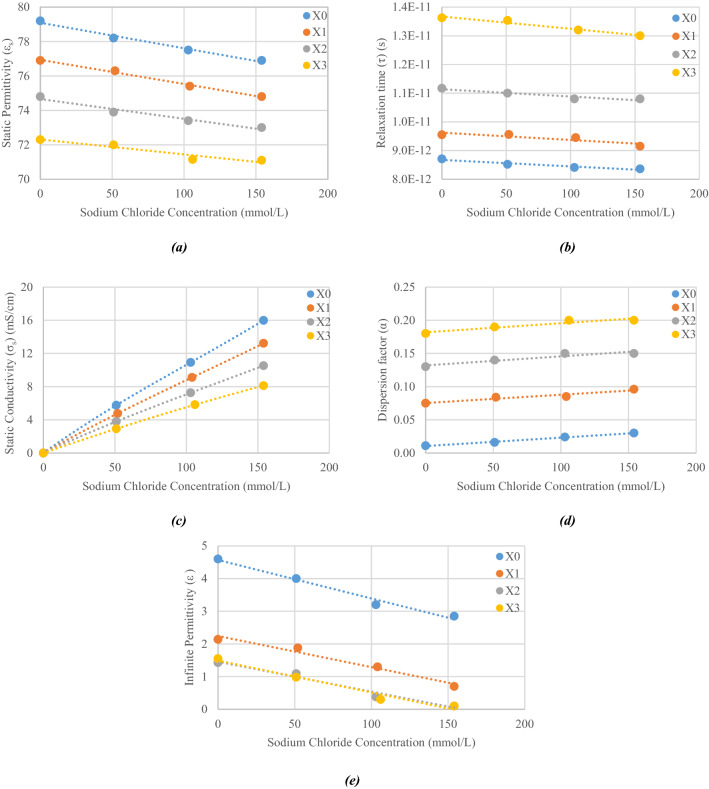
Cole–Cole Model parameters for various concentrations in sodium chloride and sucrose. X corresponds to letters A to D in Table 3. (**a**) Static permittivity, (**b**) relaxation time, (**c**) conductivity, (**d**) dispersion factor and (**e**) infinite permittivity.

In general, solutes interactions take place in ternary solutions and modify the variation laws of the parameters of binary mixtures. Variations of parameters ${\varepsilon }_{s}$, $\tau$, ${\varepsilon }_{\infty }, \alpha$ and ${\sigma }_{s}$ were plotted in Fig. 5 with respect to the concentrations of sodium chloride for the different concentrations of sucrose.

All parameters show quite linear variations depending on the sodium chloride concentration, slopes and intercepts of which depend on the sucrose concentration.

Now that the interaction effects occurring in ternary mixtures have been identified and model parameters have been extracted from measurement results, the next section will provide predictive numerical models for the variations of the model parameters regarding sodium chloride and sucrose concentrations.

#### Variational laws of the Cole–Cole model parameters for ternary mixtures

Polynomial equations were computed from the data reported in Table 3, relating each of the Cole–Cole model parameter (static permittivity, relaxation time, ionic conductivity, distribution parameter and infinite permittivity) to both the concentrations of sucrose and sodium chloride. The following equations are provided under the assumption that all the concentrations are given in mol/L, the concentrations are within the 0 to 0.154 mol/L range for sodium chloride and 0 to 0.877 mol/L for sucrose, temperature was stable and set to 23 °C.

In these conditions, static dielectric permittivity ${\varepsilon }_{stat}$ can be expressed as:

3 $${\varepsilon }_{stat} = {{\varepsilon }_{stat \,DIW} }+\left(- \,8.85{\times } {C_{{{\text{C}}_{12}{\text{H}}_{22}{\text{O}}_{11}}}}+16.20\right)\times {C}_{\text{NaCl}}+7.79 {\times} {C_{{\text{C}}_{12}{\text{H}}_{22}{\text{O}}_{11}}},$$

where ${{\varepsilon }_{stat \,DIW}}$ is the static dielectric permittivity of DI-water. The absolute error between modelled and measured static permittivity is less than 0.2.

Since static ionic conductivity strongly depends on the concentration of sodium chloride, we have chosen to model it precisely using a third-degree polynomial. So σ_S_ can be expressed as:

4 $${\sigma }_{s}=\left(-\, 825.4{\times } {C_{{\text{C}}_{12}{\text{H}}_{22}{\text{O}}_{11}}}+672.5\right)\times {C}_{NaCl}^{3}-\left(211.9{\times } {C_{{\text{C}}_{12}{\text{H}}_{22}{\text{O}}_{11}}}+214.9\right)\times {C}_{NaCl}^{2}-\left(71.5 \times {C_{{\text{C}}_{12}{\text{H}}_{22}{\text{O}}_{11}}}-120.5\right)\times {C}_{\text{NaCl}},$$

where ${\sigma }_{s}$ is expressed in mS/cm. The absolute error between modelled and measured static ionic conductivity is less than 0.12.

Relaxation time τ can be expressed as:

5 $$\tau =-\left(2.96\times {{C}_{{\text{C}}{_{12}{\text{H}}_{22}{\text{O}}_{11}}}^{2}}-0.65\times {{C}_{{\text{C}}_{12}{\text{H}}_{22}{\text{O}}_{11}}}+2.31\right){\times C}_{\text{NaCl}}+\left(4.25\times {{C}_{{\text{C}}_{12}{\text{H}}_{22}{\text{O}}_{11}}^{2}}-1.87\times {{C}_{{\text{C}}_{12}{\text{H}}_{22}{\text{O}}_{11}}}+8.68\right),$$

where τ is expressed in ps. The absolute error between modelled and measured relaxation time is less than 0.13 ps.

Dispersion parameter $\alpha$ expression is:

6 $$\alpha = \left(0.013{\times } {C_{{\text{C}}_{12}{\text{H}}_{22}{\text{O}}_{11}}}+0.125)\times {C}_{\text{NaCl}}+(0.195\times {{C}_{{\text{C}}_{12}{\text{H}}_{22}{\text{O}}_{11}}}+0.014\right).$$

The absolute error between modelled and measured value of parameter $\alpha$ is less than 0.008.

Optimization parameter *ε*_∞_ can be expressed as:

7 $${\varepsilon }_{\infty }=\left(-\, 8.45\times {{C}_{{\text{C}}_{12}{\text{H}}_{22}{\text{O}}_{11}}^{2}}+9.61\times {{C}_{{\text{C}}_{12}{\text{H}}_{22}{\text{O}}_{11}}}-11.75\right){\times C}_{NaCl}+\left(6.93\times {{C}_{{\text{C}}_{12}{\text{H}}_{22}{\text{O}}_{11}}^{2}}-9.51\times {{C}_{{\text{C}}_{12}{\text{H}}_{22}{\text{O}}_{11}}}+4.53\right).$$

The absolute error between modelled and measured value of parameter ε_∞_ is less than 0.2.

Now that the model parameters have been extracted and their variations modelled as a function of sucrose and sodium chloride concentrations, the EM response of the mixtures can be predicted whatever the solutes concentrations in the given ranges. Furthermore, by solving the inverse problem, it is possible to determine sodium chloride and sucrose concentrations in a ternary mixture as long as at least two parameters of the model are known: the static permittivity and the ionic conductivity, for example. This is the starting point to develop microwave sensors dedicated to the monitoring of concentrations in ternary mixtures, which is detailed in the next section.

### Sensor design

#### Sensing principle

As demonstrated before, extraction of concentrations can be made from Eqs. (3) and (4) as long as conductivity and real part of the static dielectric permittivity are known. However, from a single low-frequency measurement, separating effects of conductivity from those related to static dielectric permittivity is not possible without additional information: it is compulsory to obtain complementary data. In this paper, we propose to obtain this complementary information from microwave-range measurement. Indeed, as shown in the previous section, solute concentrations of ternary mixtures can be identified from microwave frequencies dielectric properties of the solutions. To achieve high sensitivities, choice was made to rely on a RF planar resonant sensor topology. In addition to great sensitivities that could be achieved, resonant sensors usually require relatively simple driving electronics and systems to perform measurements, enabling their easy integration and featuring nice portability potential.

To limit the handling of samples and to keep the number of items reduced to the minimum, the use of a single sensor employing a single sample to perform both RF and low-frequency measurements is highly advised. The idea was thus to develop a unique sensor designed to extract low-frequency conductivity and complementary information from RF-range to extract simultaneously sodium chloride and sucrose concentrations out of ternary mixtures.

To achieve conductivity measurements at low frequencies, a classical way is to design a serial capacitance between two ports and to place the Material Under Test (MUT) upon it, in contact with the electrodes. MUT affects the serial capacitance, which is thus short-circuited due to the presence and displacement of ions if the MUT is conductive. Measurement of the resulting resistivity enables extraction of the conductivity.

**Figure 6:**
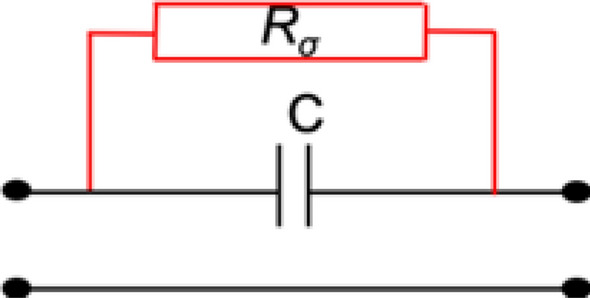
Lumped-element model of a conductivity sensor (low frequencies).

Figure 6 shows the principle of such a conductivity sensor operating at low frequencies.

In this Figure, *C* stands for the serial capacitance whereas ${R}_{\sigma }$, serial resistance, is modelling the conductive effect brought by the ions existing in the solution, ${R}_{\sigma }$ is proportional to angular frequency $\omega$ divided by the static conductivity ${\sigma }_{s}$. Thus, ${R}_{\sigma }$ is very small at low frequencies (short-circuit behaviour) and is rapidly increasing as the frequency increases (almost open-circuit behavior).

Using such a serial-capacitance sensing principle for low frequencies enables the achievement of conductivity measurements, however, it also limits the possibilities of design for the microwave sensors.

Indeed, it means that (a) the only capacitance seen at low frequencies between input and output ports should be the one under the MUT, and (b) the design should be made so that it has great interactions at RF frequencies with the MUT located over the capacitance.

Therefore, all the topologies relying on capacitively-coupled accesses or those showing direct DC input-to-output paths should be discarded.

Considering these design constraints, for the RF design, sensor topology presented in Ref.^66^ was chosen as a starting point. This sensor is composed of two facing Split Ring Resonators (SRR), also called Open-Loop Resonators (OLR), with the sample placed in the electric coupling slot area. This topology was chosen because it exhibits high sensitivities to dielectric permittivity changes such as those of glucose, and consequently those of sucrose. Furthermore, this topology is compatible with the low frequency requirements described hereinbefore.

**Figure 7:**
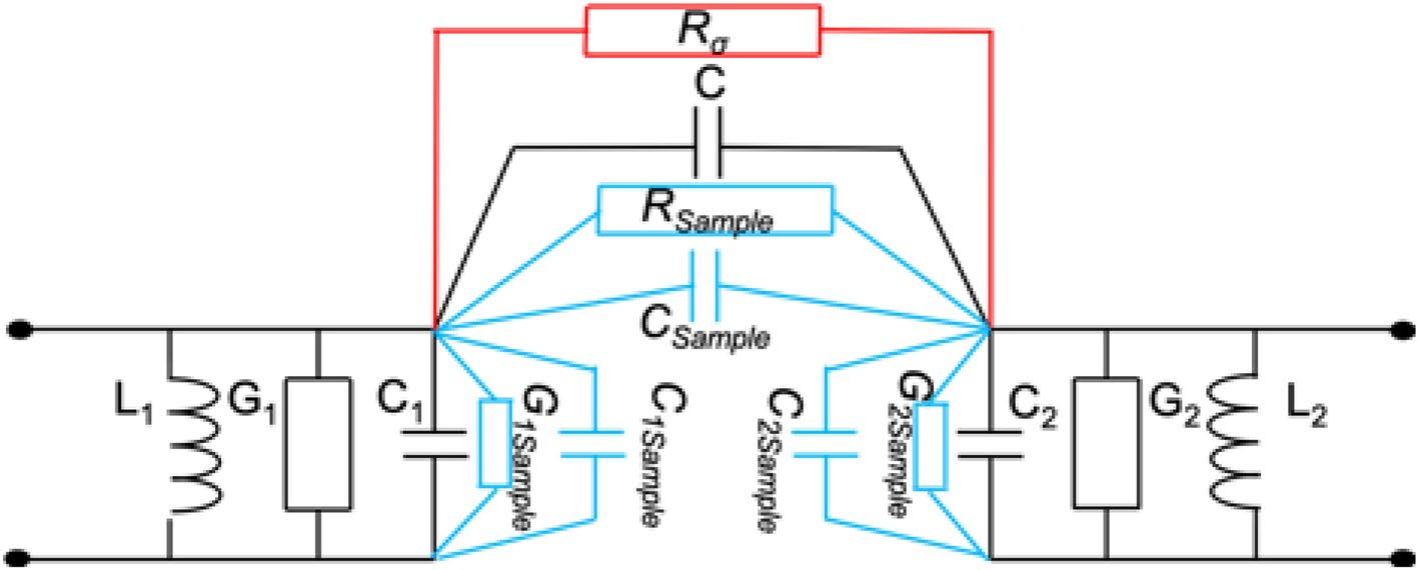
Lumped-element model of the sensor.

Sensing principle of such a topology can be modelled with the lumped-element circuit presented in Fig. 7.

In this model, ${G}_{1}, {C}_{1}, {L}_{1}$ and ${G}_{2}, {C}_{2}, {L}_{2}$ are the lumped elements modelling the first and second resonator, respectively. *C* stands for the coupling between the two resonators and finally ${G}_{1Sample}$, ${C}_{1Sample}$, ${G}_{2Sample}$, ${C}_{2Sample}$, ${R}_{Sample}$ and ${C}_{Sample}$ refer to the dielectric contributions of the sample (MUT) to each constitutive element of the sensor model. ${R}_{\sigma }$ models the conductive effect of the sample.

The sensor is thus built from two resonators coupled to each other, which are fed by input–output lines directly connected to the resonators. The sample is placed in the coupling area, at the open-ended tips of the split ring resonators (SRR). This area is chosen as it is the location where the electric field is maximum, thus the sample greatly affects the inter-resonator coupling (contributions of ${R}_{Sample}$ and ${C}_{Sample}$). On the other hand, it also affects the inner capacitance and resistance of each resonator as the sample is close to the open-ended tips of the resonators. This latter contribution of the sample over each resonator is modelled thanks to ${G}_{1Sample}$ and ${C}_{1Sample}$ for the first resonator and ${G}_{2Sample}$ and ${C}_{2Sample}$ for the second resonator.

One should note that if the physical implementation is strictly symmetrical yielding identical resonators, values with indices 1 and 2 are equal (${G}_{1}= {G}_{2}$, ${C}_{1}= {C}_{2}$, ${L}_{1}= {L}_{2}$, ${G}_{1Sample}= {G}_{2Sample}$ and ${C}_{1Sample}= {C}_{2Sample}$).

It is also worthwhile to note that for very low frequencies, resonators are not resonating, and this model can be simplified, to a model alike the one presented in Fig. 6. Reversely, model in Fig. 7 is relevant for a microwave sensor under the assumption that ${R}_{\sigma }$ is high enough (open-circuited behaviour) to ensure a good sensitivity with respect to ${R}_{Sample}$ and ${C}_{Sample}$. This is related to the choice of the working frequency and the design geometry, which will be presented in the next section. Once the frequency range is chosen, variations in the dielectric properties of the samples ($\varepsilon^{\prime}$ and $\varepsilon^{\prime\prime}$), which are related to sodium chloride and sucrose concentrations, will modify ${G}_{1Sample}$, ${C}_{1Sample}$, ${G}_{2Sample}$, ${C}_{2Sample}$, ${R}_{Sample}$ and ${C}_{Sample}$, and consequently the associated electrical response of the sensor, which is observed through its scattering parameters.

#### Sensor design

Once the sensing topology is chosen, the next step toward the design of the sensor is to determine the targeted frequency range of operation for the microwave part. Different effects have to be considered to do so.

On one hand, microwave operating frequency should be high enough to evacuate the conductive effects of sodium chloride as shown in Fig. 2, which means that frequencies lower than 5 GHz should be discarded.

On the other hand, high frequencies (> 10 GHz) are not suitable because the real part of the permittivity for all water-based solutions is decreasing toward high frequencies. Using those high frequencies would mean low values for the real part of the permittivity (see Fig. 4), which would lead to less concentration of the electric field distribution within the sample, which is not favorable for sensing purposes. Additionally, the imaginary part of the dielectric permittivity is reaching a maximum around 12 GHz, which means increased losses at these frequencies.

To identify the best frequency range for sensing purposes, maximum variations of the real part of the dielectric permittivity obtained thanks to the wideband EM characterizations presented in “Ternary mixtures involving water, sodium chloride and sucrose” section were computed. Indeed, sensitivity of the sensor is mainly linked (although not exclusively) to the variations of ${C}_{1Sample}$, ${C}_{2Sample}$, and ${C}_{Sample}$, which are mainly related to changes of the real part of the permittivity of solution.

**Figure 8:**
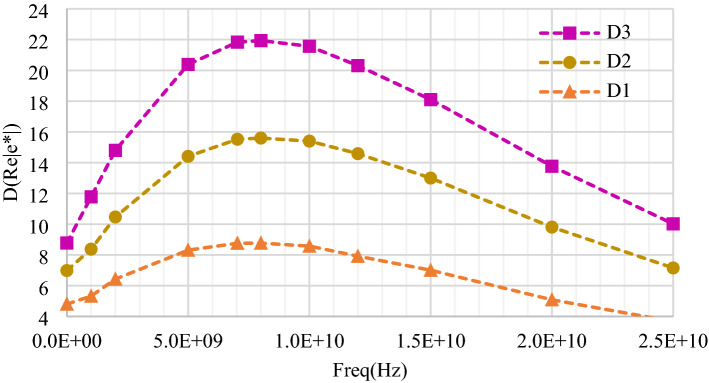
Δ(Re(ε*))(f), Maximum difference in the real part of the permittivity as a function of frequency.

In order to identify the best operating frequency, the difference between the permittivity real part of several solutions and those of DI water (DIW) was measured at each frequency for all solutions. The resulting difference ($\Delta \varepsilon^{\prime}\left( f \right) = \varepsilon_{DIW} \left( f \right) - \varepsilon_{{\left\{ {Samples} \right\} }} \left( f \right)$ is plotted as a function of frequency (*f)* in Fig. 8 for three solutions.

This plot shows that maximum difference of the real part of the permittivity is reached around 8 GHz.

Similarly, ${G}_{1Sample}$, ${G}_{2Sample}$, ${R}_{Sample}$ are mainly linked to the values of the imaginary part of the permittivity (Fig. 3), which should be chosen as low as possible to minimize losses, but whose variations might also be interesting for sensing purposes.

To trade-off between all these effects, an operating frequency around 7 GHz was chosen. At this frequency, the real part of the dielectric permittivity can be expressed as:

8 $$\varepsilon \left(7 \text{GHz}\right) ={\varepsilon }_{DIW}\left(7 \text{GHz}\right)+\left(-\,9.39\times {C}_{\text{NaCl}}+23.78\right) {{C}_{{\text{C}}_{12}{\text{H}}_{22}{\text{O}}_{11}}}+15.58 \times {C}_{\text{NaCl}},$$

where ${\varepsilon }_{DIW}\left(7 \text{GHz}\right)$ is the real part of the dielectric permittivity of DI water at 7 GHz and ${\varepsilon }_{7 \text{GHz}}$ stands for the real part of the dielectric permittivity, at 7 GHz, of a mixture with a given sodium chloride concentration $\left({C}_{\text{NaCl}}\right)$ and a given sucrose concentration $\left({{C}_{{\text{C}}_{12}{\text{H}}_{22}{\text{O}}_{11}}}\right)$ in mol/L, all the other parameters and assumptions being the same as in “Ternary mixtures involving water, sodium chloride and sucrose” section. The absolute error between modelled and measured real part of permittivity at 7 GHz is less than 0.2. At this point, it is worthy to note the increase of sensitivity to sucrose concentration at 7 GHz with respect to that presented in Eq. (3) at DC, reinforcing the relevancy to choose RF range for the operating frequency.

To implement the sensor, a 0.8 mm-thick RT Duroid 5880 substrate with a relative dielectric permittivity of 2.2 and a loss tangent of 0.0009 was chosen.

Concerning the sample, it must be placed in the area where the electrical field is maximum, i.e. in the slot between the two resonators. Additionally, the liquid sample has to be in contact with the metal strips to allow for conductivity measurement at low frequencies. Consequently, a bottomless sample holder was made thanks to a 3D-printer. This annular-wall shaped piece was then stuck onto the designed circuit. Material used to achieve the sample-holder is RS-F2-GPWH-04 from FormLabs^®^, once cured, its expected relative dielectric permittivity is 3.55 and loss tangent is 0.01. The glue to stick the sample holder and ensure a proper sealing is Loctite™ EA 3421from Henkel^®^.

A simulated operating frequency around 7 GHz when loaded with samples was achieved with dimensions of *L* = 10 mm and *l* = 5.4 mm. The remaining parameters (*w,* width of the SRR lines, equal to 1.8 mm), (*g,* slot between the two resonators, equal to 1.2 mm) and (*e,* open-end gap of the SRR equal to 2 mm) did show remarkable influence upon the sensitivities (both to sodium chloride and sucrose concentrations), and had to be tuned in a recursive manner to obtain the best trade-off. Finally, input/output lines were designed to show a characteristic impedance of 50 ohms (T_L width_ = 2.474 mm), their location being tuned to insure an appropriate coupling (Z = *5* mm).

**Figure 9:**
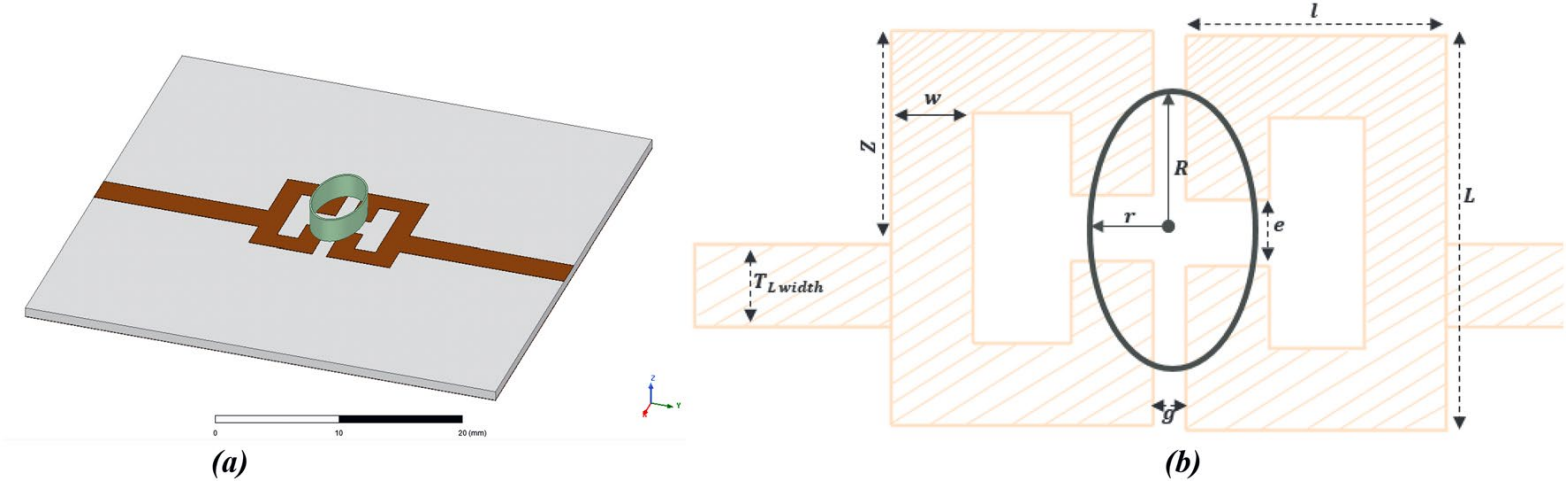
(**a**) 3D model and (**b**) layout of the designed sensor.

The layout of the resulting sensor and its dimensions are shown in Fig. 9.

Size of the bottomless sample holder was tuned to R = 3.4 mm × r = 2.04 mm, which correspond to an estimated sample volume of 43.56 μL*.* The MUT container is located in the area where the electric field is maximum, and centered to ensure a symmetrical design.

**Figure 10:**
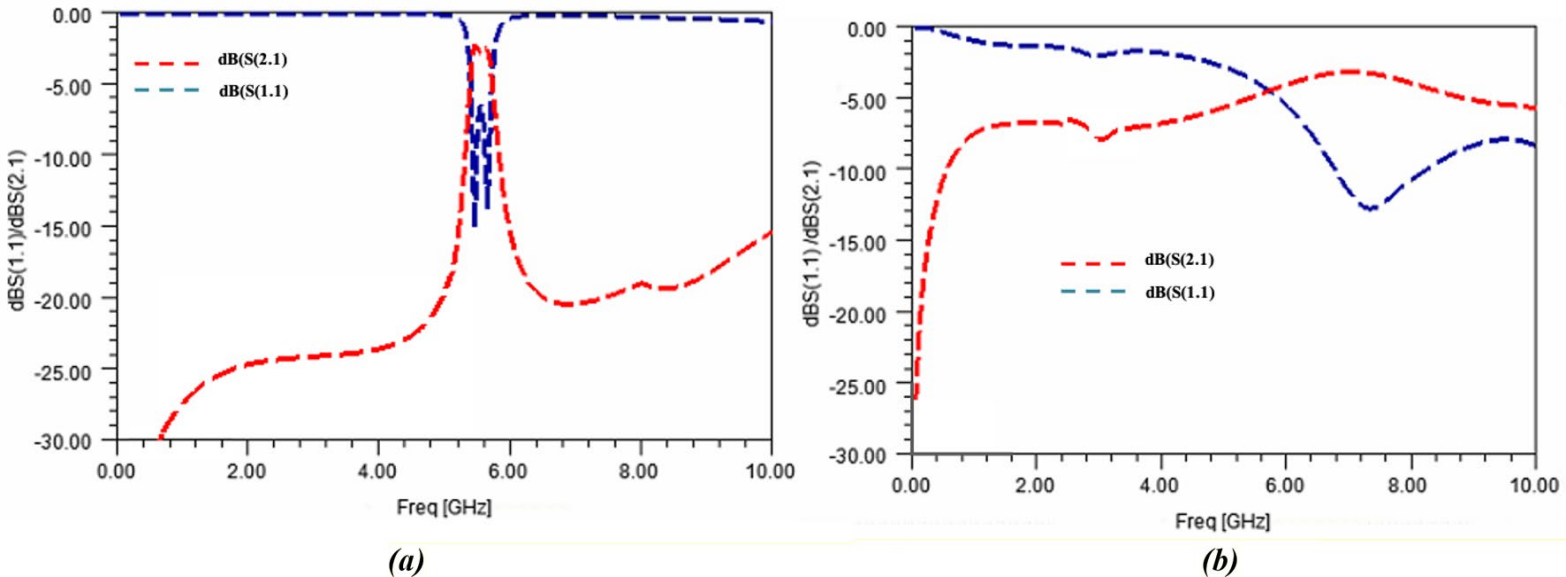
S-parameters obtained with full-wave simulations of the designed sensor (**a**) loaded with air and (**b**) with DI-water.

Full wave simulations (run with HFSS™ software from ANSYS^®^) of the sensor with air and DI-water as MUTs are shown in Fig. 10. One should note that DI-water was modelled at this stage with the Cole–Cole model parameters extracted from the above-mentioned wide-band characterization of the A0 sample. Consequently, in the simulations, the DI-water, considered at room temperature, was modelled with a static permittivity ${\varepsilon }_{stat}$ of 79.2, an infinite permittivity ${\varepsilon }_{\infty }$ of 4.6 and a relaxation time $\tau$ of 8.71 ps.

#### Adjustment of the sensor model

**Figure 11:**
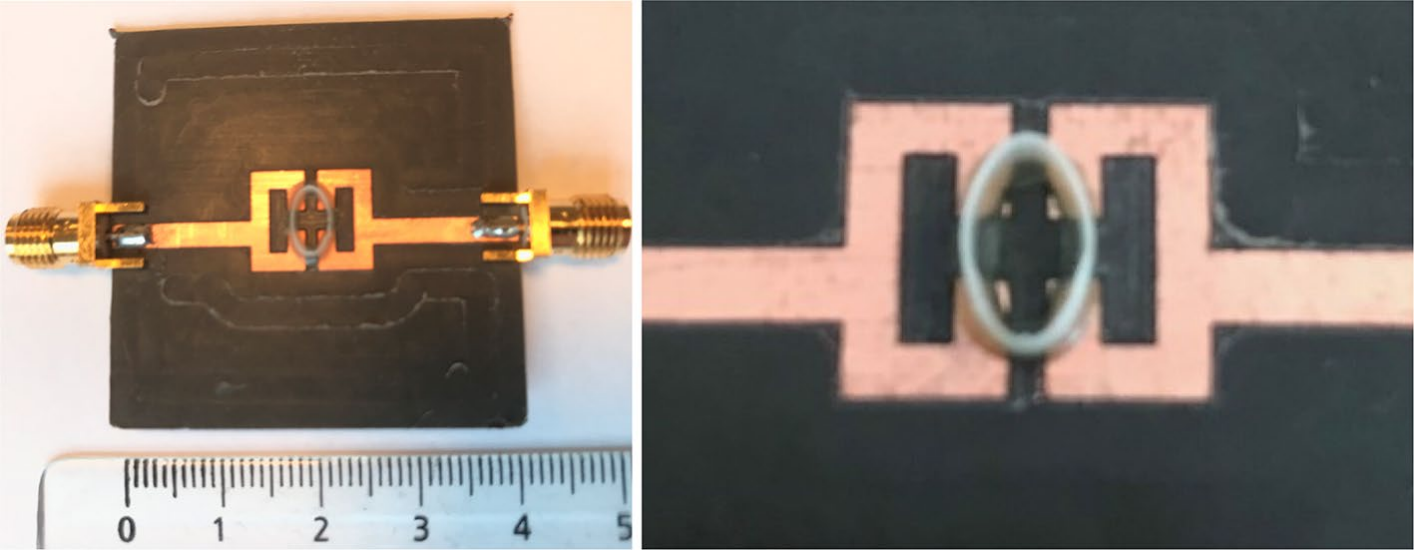
Fabricated sensor (with a centimetric scale) and zoomed-in view of the sensing area.

The sensor parts were realized and assembled; a photography of the realized sensor is shown in Fig. 11.

**Figure 12:**
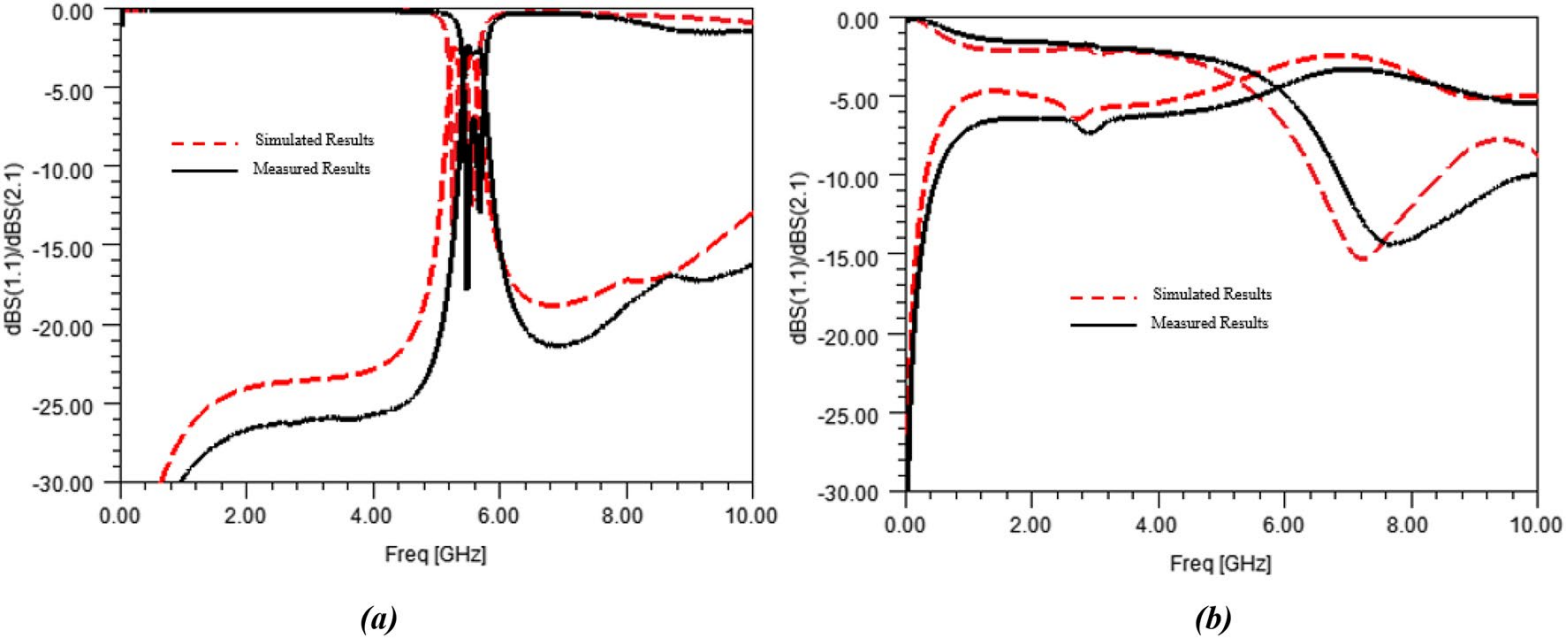
Simulated and measured S-parameters of the sensor with the container filled in with (**a**) air and with (**b**) DI-water.

The sensor was then measured using a properly-calibrated Vector Network Analyzer (Rhode & Schwarz^®^ ZVA 67). Simulations and measurements of the sensor with an empty container (i.e. air as MUT) are shown in Fig. 12a together with the simulated and measured results when the container is filled with DI-water (A0 solution), which are presented in Fig. 12b.

As it can be noted from Fig. 12, simulations of the initial model of the sensor are slightly different from measurements. It means that the proposed 3D-model of the sensor does not fit exactly the measurements of the realized sensor, the differences, mainly a frequency shift, can be attributed to inaccurate material description or technological dispersions. Concerning those latter ones, they can be related to machine tolerances or deviations, which could impact metal etching or container size. Technological dispersions may also be due to the manmade gluing of the container, entailing uncertainties upon the size of the final glue tie and the container position.

To consider all those dispersions, retrofitting electromagnetic simulations were run to refine the sensor model trying to make its simulations fit both the air and DI-water measurements. Sensitive design parameters were identified and tuned. The modified dimensions are g = 1.6 mm and size of the bottomless container R = 3.31 mm × r = 2.06 mm. The glue tie model and dimensions were also slightly modified, relative changes on their parameters being lower than 5%.

Concerning the material properties tuning, materials of the sensor only were tuned, MUT models (air or DI-water) were kept untouched during this retrofit step, and simulations yield a change on the relative dielectric permittivity of the substrate only, moving from 2.23 to 2.1.

**Figure 13:**
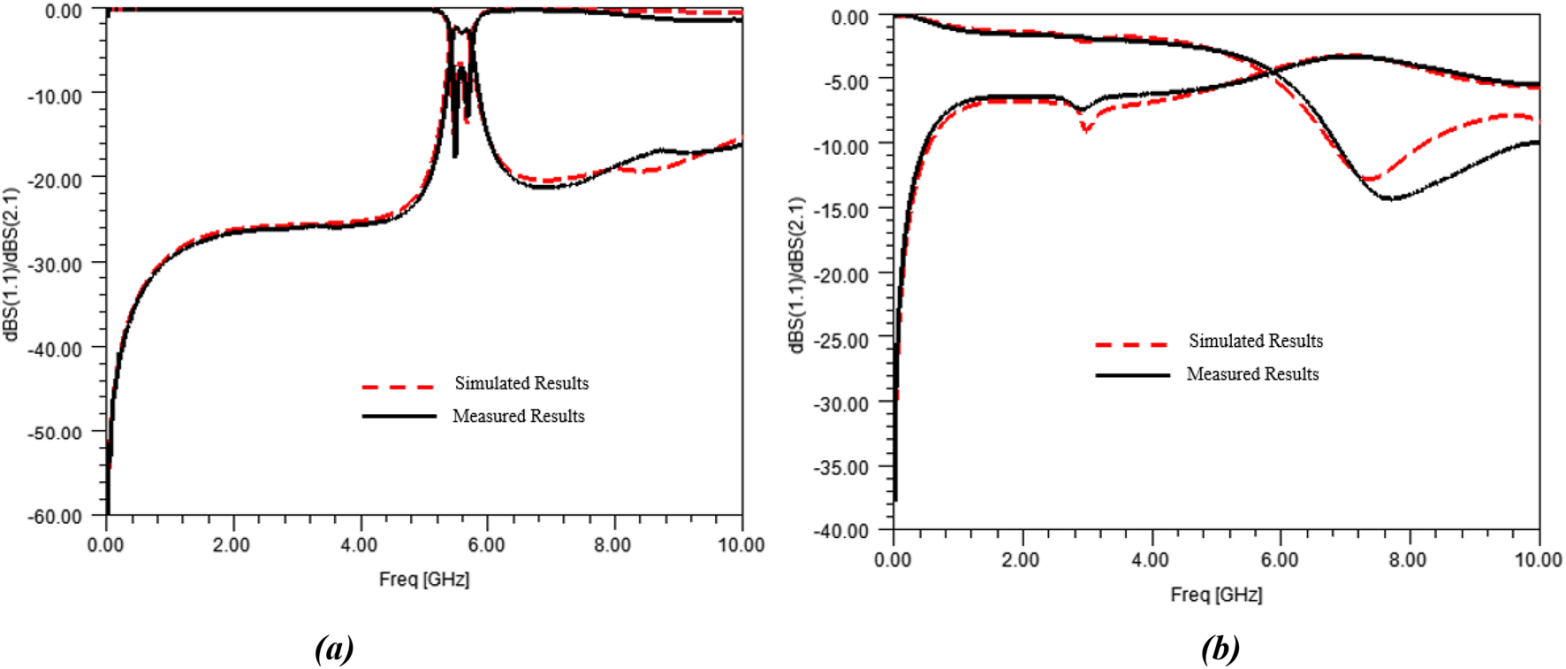
Measured and retrofitted (EM simulations) S-parameters of the sensor with the container filled in with (**a**) air and with (**b**) DI-water.

Finally, the corrected model simulations are plotted in Fig. 13 together with the measured results.

#### Evaluation of sensor sensitivities from simulated data

To determine the sensing capability of the proposed design, full-wave simulations of the updated sensor were run in HFSS™ with various samples. These latter were modelled thanks to a Cole–Cole model with the parameters provided in “Dielectric characterization and modelling of aqueous solutions” section (Table 3). Broadband simulations were conducted but two frequency ranges were particularly scrutinized: the first being at low frequencies (between 10 MHz and 2 GHz) to track conductive effects while the second is located at higher frequencies (around the 7 GHz), following explanations given in “Dielectric characterization and modelling of aqueous solutions” section.

**Figure 14:**
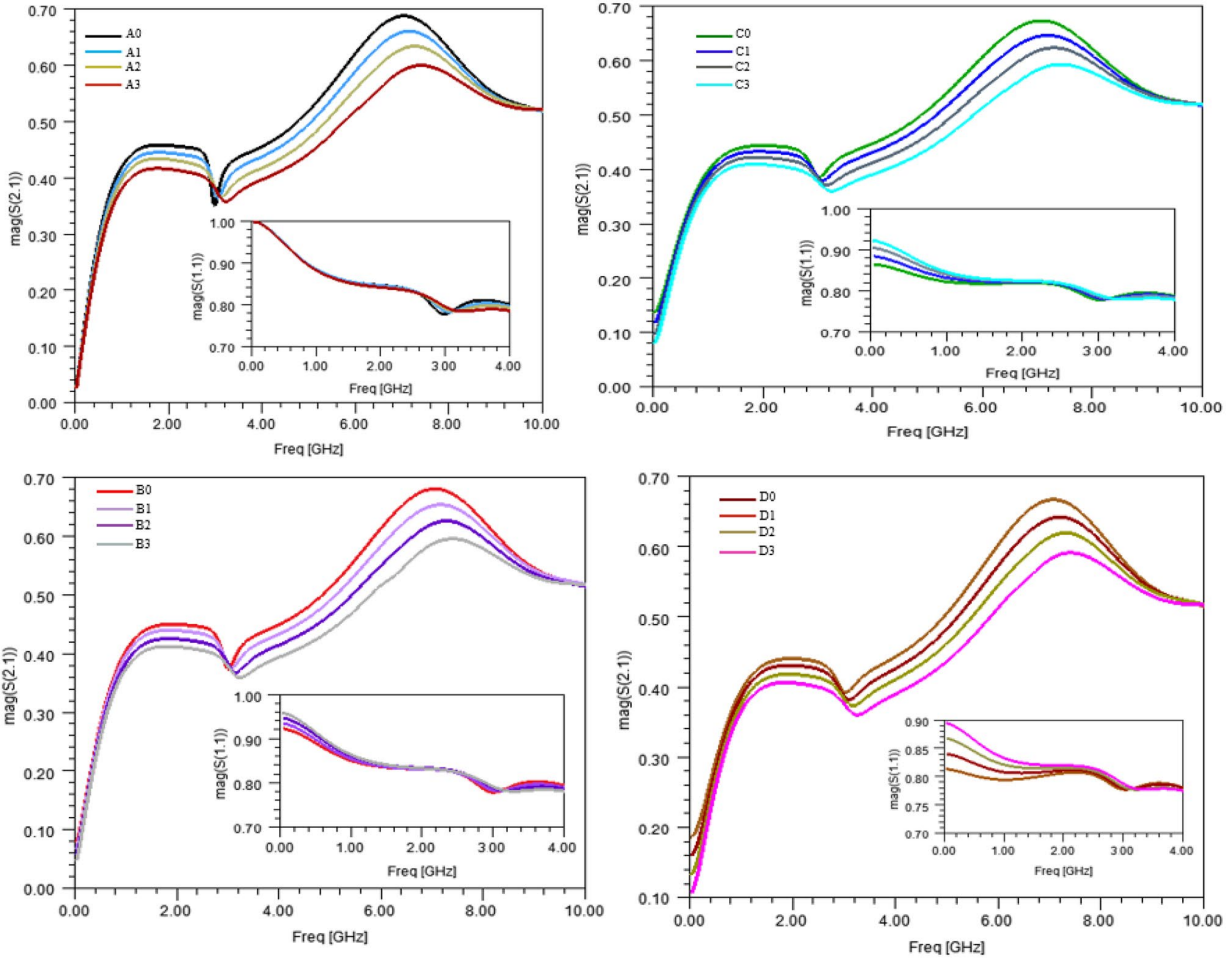
Simulated transmitted (S21) and reflected (S11) S-parameters of the sensor with various modelled samples.

Figure 14 shows the simulated transmission S-parameter for the sensor with various modelled samples.

One can note the distinctive effects at low frequencies occurring when the modelled samples exhibit sodium chloride concentration changes (modelled samples A0, B0, C0, D0) whereas, as foreseen, samples modelling changes in sucrose concentration impact the transmitted S-parameter in the microwave range.

From these EM simulations, and aimed to assess sensor performances, several sensing magnitudes could be chosen, resulting in a trade-off between sensitivity, ease of measurement, data extraction and computation easiness, and error level related to measurement noise floor^67^.

Concerning low frequencies, since the objective is to extract the conductivity, sensing magnitudes could be either the magnitude of the transmitted S-parameter (S_21_) or the reflected S-parameter (S_11_). We chose the latter one (in natural value) because of its higher level and better sensitivity, and the frequency of 100 MHz was chosen to extract this sensing parameter.

In the microwave range, as detailed in Ref.^66^, several indicators could be chosen as sensing parameters, such as for instance, magnitude of the transmission S-parameter (S_21_) or coupling coefficient *K.* The sensor being composed of two coupled resonators, its electrical response is alike a second order filter, and following the design choices detailed in “Sensor design” section, the coupling between the two resonators is strong, preventing the appearance of two distinct peaks in the transmitted scattering parameter. As the sample affects not only the mutual coupling (*C*_*Sample*_ and *R*_*Sample*_, see Fig. 7) and consequently the bandwidth and insertion losses of the second order filter, but also intrinsic resonant frequency and Q-factor of each resonator through *C*_*1Sampl*e_, *C*_*2Sample*_, *R*_*1Sample*_, and *R*_*2Sample*_, we chose to define a sensing parameter, alike an unloaded Q-factor, but for a second-order filter. Definition of this dimensionless factor called Sensing Ratio (*SR*) is given hereafter:

9 $$SR=\frac{\left(\frac{{f}_{c}}{BW}\right)}{\left(1-\left|{S}_{21}\left({f}_{c}\right)\right|\right) },$$

where *BW* is the bandwidth computed from 1 dB fall from S_21_ maximum (instead of the commonly-used 3 dB fall, which cannot be computed for high sucrose concentrations, because of losses), *f*_*c*_ is the central resonant frequency and $\left|{S}_{21}\left({f}_{c}\right)\right|$ is the magnitude (in natural value) of the transmission S-parameter at the central frequency.

Table 4 summarizes values of those two sensing parameters ($\left|{S}_{11}\left(100 \,\text{MHz}\right)\right|$ & *SR*) for simulations performed with each sample modelled with the parameters provided in Table 3.

**Table 4:** Sensing parameters for each modelled sample, extracted from EM simulations.

Modelled sample	Simulated $\left|{S}_{11}\left(100 \,\text{MHz}\right)\right|$	Simulated *SR*
A0	0.996	9.818
B0	0.921	9.517
C0	0.863	9.255
D0	0.813	8.986
A1	0.996	8.781
B1	0.933	8.531
C1	0.883	8.302
D1	0.839	8.118
A2	0.996	7.703
B2	0.945	7.530
C2	0.903	7.334
D2	0.866	7.176
A3	0.996	6.508
B3	0.956	6.395
C3	0.920	6.311
D3	0.893	6.247

**Figure 15:**
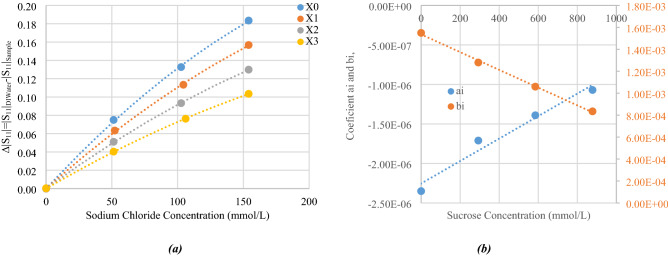
(**a**) Simulated values of |S11 (100 MHz)| as a function of sodium chloride concentration, and (**b**) values of the coefficients a_i_ and b_i_ of the polynomial approximation as a function of sucrose concentration.

From Table 4, simulated value of $\left|{S}_{11}\left(100 \,\text{MHz}\right)\right|$ for each sample was subtracted from that of the water (A0, with a $\left|{S}_{11}\left(100 \,\text{MHz}\right)\right| = 0.996$) and plotted as a function of sodium chloride concentration. The resulting plot can be seen in Fig. 15a, where sodium chloride concentration is given in x-axis and expressed in mmol/L.

Several groups of points are plotted, which stand for various sucrose concentrations, with *X*0, *X*1, *X*2, *X*3 representing sucrose concentrations of 0, 292, 584 and 877 mmol/L, respectively. First order approximations are then extrapolated from the plotted points, yielding a second order polynomial equation with a regression coefficient better than 0.9997. Slope of each line represent the sensitivity of $\left|{S}_{11}\left(100 \,\text{MHz}\right)\right|$ of the designed sensor to sodium chloride.

At this point, one should note that this is fully consistent with the behavior related to the conductivity in the measured samples evidenced in Fig. 5c, demonstrating the relevancy of the design sensor on one hand and of the chosen sensing parameter on the other hand.

Figure 15a shows the impact of sucrose concentration over the $\left|{S}_{11}\left(100 \,\text{MHz}\right)\right|$ sensitivity, which could also be considered as cross-sensitivity of sucrose concentration over sodium chloride concentration sensitivity of the sensor. Polynomial equations were computed from the data reported in Fig. 15. The following equations are provided under the assumption that all the concentrations are given in mol/L and the temperature is stable and set to 23 °C.

Since values of $\left|{S}_{11}\left(100 \,\text{MHz}\right)\right|$ strongly depends on the concentration of sodium chloride, we have chosen to model it precisely using a second-order polynomial. When standardized with respect to that of DI water, $\left|{S}_{11}\left(100 \,\text{MHz}\right)\right|$ of a given sample can be expressed as:

10 $$\left|{S}_{11}\left(100 \,\text{MHz}\right)\right|={\left|{S}_{11}\left(100 \,\text{MHz}\right)\right|}_{DI water}-\left[{a}_{i} {C}_{\text{NaCl}}^{2}+ {b}_{i} {C}_{\text{NaCl}}\right].$$

Values of these coefficients a_i_ and b_i_ are summarized in Fig. 15b as a function of sucrose concentrations. This plot shows the impact of sucrose concentration over the $\left|{S}_{11}\left(100 \,\text{MHz}\right)\right|$ sensitivity. Again, a linear approximation was made, which led to a regression coefficient of R^2^ = 0.966, showing a good linearity. Expression of these coefficients are:

11 $${a}_{i}=1.42\times {{C}_{{\text{C}}_{12}{\text{H}}_{22}{\text{O}}_{11}}}-2.25,$$

12 $${b}_{i}=-\,8.08\times {10}^{-1}\times {{C}_{{\text{C}}_{12}{\text{H}}_{22}{\text{O}}_{11}}}+1.53.$$

**Figure 16:**
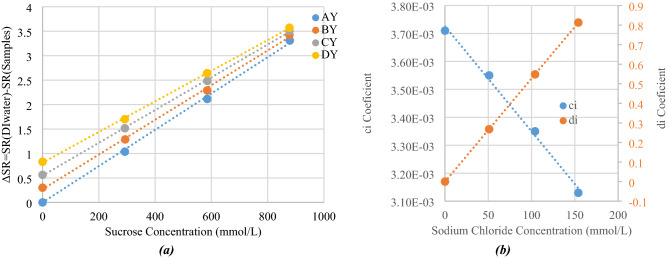
(**a**) Simulated values of SR as a function of sucrose concentration, and (**b**) slope c_i_ and origin d_i_ of the first-order approximations as a function of sodium chloride concentration.

The same approach was then performed concerning *SR* from Table 4, the computed *SR* value for each modelled sample is subtracted from that of DI-water (for A0, *SR* = 9.818). These *SR* standardized with respect to that of DI-water are then plotted as a function of sucrose concentrations for various sodium chloride concentrations in Fig. 16a. In this Figure, *AY, BY, CY, DY* represent sodium chloride concentrations of 0, 51, 103 and 154 mmol/L, respectively (and various sucrose concentrations).

First order (lines) approximations are then extrapolated from the plotted points, yielding a linear equation:

13 $${SR}_{Samples}={SR}_{DIW}-\left({c}_{i} {{C}_{{\text{C}}_{12}{\text{H}}_{22}{\text{O}}_{11}}}+{d}_{i}\right),$$

with a regression coefficient better than 0.9987. Slopes of each line represent the simulated sensitivity of *SR* of the designed sensor to sucrose concentration variations.

Figure 16 shows that the Sensing Ratio, which is alike a Q-factor computed at microwave frequencies from the S21 level, the central frequency and the bandwidth, is decreasing when sucrose concentration increases. This can be explained through the inverse behavior with respect to the relaxation time $\tau$ and the dispersion parameter $\alpha$, which are both increasing when sucrose concentrations increase (Figs. 4, 5b,d).

Values of the slopes (coefficient *c*_*i*_) and origins (coefficient *d*_*i*_) are summarized in Fig. 16b as a function of sodium chloride concentration. This plot shows the impact of sodium chloride concentration over *SR* sensitivity, which represents the cross-sensitivity of sodium chloride concentration over sucrose concentration sensitivity for the designed sensor.

A linear approximation with a regression coefficient of R^2^ = 0.995 was made. Expression of these coefficients are:

14 $${c}_{i}=- 3.74\times {C}_{\text{NaCl}}+3.73,$$

15 $${d}_{i}=5.27\times {C}_{\text{NaCl}}.$$

With sodium chloride concentration $\left({C}_{NaCl}\right)$ and sucrose concentration $\left({{C}_{{\text{C}}_{12}{\text{H}}_{22}{\text{O}}_{11}}}\right)$ expressed in mol/L.

Now that sensing magnitudes $\left|{S}_{11}\left(100 \,\text{MHz}\right)\right|$ and *SR* have been defined and their sensitivities with respect to sodium chloride and sucrose concentrations have been analyzed from simulated results, next section will focus on the retrieval of sample concentrations from measured electrical responses of the sensor, which relies on solving the inverse problem for Eqs. (10) and (13).

### Experimental assessment of the sensor

Measurements of the realized sensor filled in with samples were then performed. The data were measured in the frequency range of 10 MHz–15 GHz, with a resolution of 9.37 MHz (1601 points) on a properly-calibrated Rhode & Schwarz® ZVA 67 VNA.

**Figure 17:**
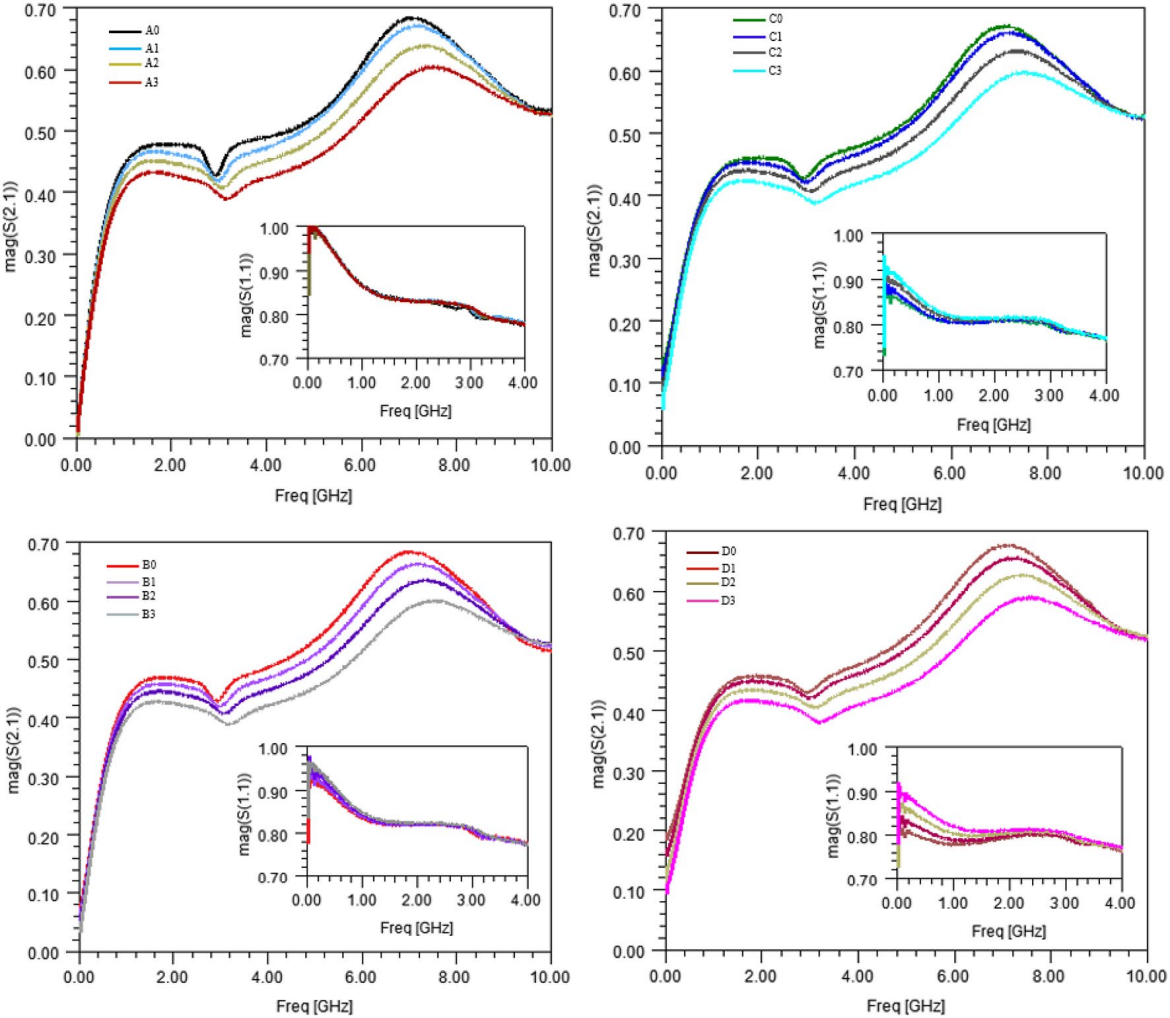
Measured transmitted (S21) and reflected (S11) parameters of the sensor with various samples.

A precision micropipette (Gilson PIPETMAN^®^ Classic P200, resolution of 0.1 μL) was used to fill the container with 43.6 μL, exactly, of each sample prepared as explained in “Ternary mixtures involving water, sodium chloride and sucrose” section (Table 5). After each sample measurement, a careful double rinsing procedure based on ethanol and DI-water was applied. It was followed by a compressed-air drying step before moving on to the next sample measurement. All measurements were made at a room temperature of 23° C. Measurement results are presented in Fig. 17.

**Table 5:** Concentrations extracted from the measurements using Eqs. (10)–(15).

Sample	Measured $\left|{S}_{11}\left(100 \,\text{MHz}\right)\right|$	Measured *SR*	Extracted sodium chloride concentration (mmol/L)	Extracted sucrose concentration (mmol/L)
A0	0.999	9.235	–	–
B0	0.919	8.971	56	0
C0	0.861	8.681	106	0
D0	0.813	8.426	157	0
A1	0.999	8.213	0	274
B1	0.933	8.012	54	266
C1	0.883	7.796	103	268
D1	0.839	7.568	156	269
A2	0.999	7.090	0	577
B2	0.945	6.926	54	575
C2	0.903	6.808	103	564
D2	0.866	6.671	155	556
A3	0.999	6.044	0	857
B3	0.958	5.935	52	858
C3	0.920	5.820	108	857
D3	0.891	5.735	158	851

At this point, one should note that broadband measurements are shown for the exhaustivity sake, but retrieval of the concentrations are based on the measured S-parameters at 100 MHz on one hand, and on a frequency band located between 6 and 8 GHz on the other hand.

Table 5 presents the values of the return loss level at 100 MHz ($\left|{\text{S}}_{11}\left(100\text{ MHz}\right)\right|$) and the Sensing Ratio (*SR*, computed from Eq. (7) definition) directly extracted from the measurements shown in Fig. 17.

Then, from those measured values of $\left|{S}_{11}\left(100 \,\text{MHz}\right)\right|$ and *SR* on one hand, and Eqs. (10)–(15) on the other hand, solving the inverse problem yields retrieved concentrations for sucrose and sodium chloride. Those retrieved concentrations, extracted from measurements, are also reported in Table 5.

This table shows that it is possible to extract both sucrose and sodium chloride concentrations from the measurement of a single sensor. However, in order to further evaluate the error made while computing the concentrations and thus the overall sensor accuracy, those retrieved concentrations have to be compared to the real ones. Table 6 summarizes, for all samples, effective concentrations (labelled “Real Concentrations”, which are the same data than those in Table 5) and retrieved concentrations (i.e. obtained by solving the inverse problem of Eqs. (10)–(15) upon measured $\left|{S}_{11}\left(100 \,\text{MHz}\right)\right|$ and *SR*)*.* One should note that the inverse problem solving has led to unrealistic negative (yet very close to zero) values for the concentrations, which have consequently been set to zero. The relative differences for each compound concentration together with the quadratic error were also computed and reported in the same table.

**Table 6:** Summary of the real and extracted concentrations and their relative differences.

Sample	Sodium chloride			Sucrose			Quadratic error (%)
	Real concentration (mmol/L)	Extracted concentration (mmol/L)	Relative difference (%)	Real concentration (mmol/L)	Extracted concentration (mmol/L)	Relative difference (%)	
A0	0	0	0	0	0	0	0
B0	51	56	+ 9.8	0	0	0	9.8
C0	103	106	+ 2.9	0	0	0	2.9
D0	154	157	+ 1.9	0	0	0	1.9
A1	0	0	0	293	274	− 6.5	6.5
B1	51	54	+ 5.8	294	266	− 9.5	11.1
C1	104	103	+ 1.0	293	268	− 8.5	8.5
D1	154	156	+ 1.3	292	269	− 7.8	7.9
A2	0	0	0	585	577	− 1.4	1.4
B2	51	54	+ 5.8	585	575	− 1.7	6.0
C2	103	103	0	586	564	− 3.8	3.8
D2	154	155	+ 0.6	585	556	− 4.9	4.9
A3	0	0	0	879	857	− 2.5	2.5
B3	51	52	+ 1.9	877	858	− 2.1	2.8
C3	106	108	+ 1.9	877	857	− 2.3	2.9
D3	154	158	+ 2.6	877	851	− 2.9	3.8

Quadratic error is computed thanks to the formula:

16 $${\sigma }_{quadratic}=\sqrt{{\left({\Delta C}_{\text{NaCl}}\right)}^{2}+{\left({\Delta} {C_{{\text{C}}_{12}{\text{H}}_{22}{\text{O}}_{11}}}\right)}^{2} .}$$

Analysis of this table shows that the extracted values for concentrations are consistent with the real ones, the relative error on each compound concentration being smaller than 9.8% for the sodium chloride and 9.5% for the sucrose. Average quadratic error over the set of measured samples is 11.1%.

These results show that the study proposed in this paper enables the detection of two distinct compounds in aqueous mixtures with a single sensor, yielding an overall error over species concentrations that does not exceed 11.1%. This validates the proposed models and the choices made on the scientific approach: modelling, sensor design and sensing parameters. This also substantiates the concept of an RF-based bi-parameter sensor, which has never been previously proposed, to the best of our knowledge, in the scientific literature. However, errors presented in Table 6 could be detrimental to industrial applications where high accuracies are seeked and consequently, these errors or inaccuracies should be analyzed to identify their causes.

As described hereinbefore, the proposed study involves numerous steps and modelling stages, each of them introducing potential errors.

To evaluate the accuracy of the proposed method and to identify the contribution to the error of the steps of the approach, we have decided to extract predicting equations directly from the measurements, thus evacuating errors related to the modelling steps. Alike Eqs. (10) and (13) that were determined from simulated data, Eqs. (17) and (20) were extracted from the measurements results provided in Fig. 17.

The obtained variational laws for the sensing parameters extracted from measurements are:

17 $$\left|{S}_{11}\left(100 \,\text{MHz}\right)\right|={\left|{S}_{11}\left(100 \,\text{MHz}\right)\right|}_{H2O}-\left[{a}_{i}{ C}_{\text{NaCl}}^{2}+{b}_{i} {C}_{\text{NaCl}}\right],$$

18 $$\text{with }{a}_{i}=2.29\times {{C}_{{\text{C}}_{12}{\text{H}}_{22}{\text{O}}_{11}}}-2.94,$$

19 $$\text{and }{b}_{i}=-0.93\times {{C}_{{\text{C}}_{12}{\text{H}}_{22}{\text{O}}_{11}}}+1.65,$$

20 $$\text{and }{SR}_{Samples}={SR}_{DIW}-\left(-3.71\times {C}_{\text{NaCl}}\times {C}_{{C}_{12}{H}_{22}{O}_{11}}+3.65\times {{C}_{{\text{C}}_{12}{\text{H}}_{22}{\text{O}}_{11}}}+{5.08\times C}_{\text{NaCl}}\right),$$

with sodium chloride concentration $\left({C}_{\text{NaCl}}\right)$ and sucrose concentration $\left({{C}_{{\text{C}}_{12}{\text{H}}_{22}{\text{O}}_{11}}}\right)$ expressed in mol/L.

Then, from those Eqs. (17) and (20), solving the inverse problem from the measured data lead to the values proposed in Table 7.

**Table 7:** Summary of extracted concentrations using equations derived from measured results (Eqs. (17) and (20)).

Sample	Sodium chloride			Sucrose			Quadratic error (%)
	Real concentration (mmol/L)	Extracted concentration (mmol/L)	Relative difference (%)	Real concentration (mmol/L)	Extracted concentration (mmol/L)	Relative difference (%)	
A0	0	0	0	0	0	0	0
B0	51	53	+ 3.9	0	0	0	3.9
C0	103	103	0	0	11	N/A	0
D0	154	156	+ 1.3	0	6	N/A	1.3
A1	0	0.0	0	293	280	− 4.4	4.4
B1	51	52	+ 2.0	294	279	− 5.1	5.4
C1	104	100	− 3.8	293	284	− 3.1	4.9
D1	154	154	0	292	287	− 1.7	1.7
A2	0	0.0	0	585	588	+ 0.5	0.5
B2	51	53	+ 3.9	585	591	+ 1.0	4.0
C2	103	101	− 1.9	586	585	− 2.0	2.8
D2	154	153	− 0.6	585	580	− 0.9	1.1
A3	0	0.0	0	879	874	− 0.6	0.6
B3	51	52	+ 1.9	877	878	+ 0.1	1.9
C3	106	107	+ 0.9	877	883	+ 0.7	1.1
D3	154	156	+ 1.3	877	882	+ 0.6	1.4

This table shows a notable improvement in the Relative Difference values for sucrose concentrations and slight improvement concerning sodium chloride Extracted Concentrations with respect to Real Concentrations. It is worthwhile to note that the higher errors presented in Table 7 are related to the same samples than those exhibiting highest error level in Table 6. Improvements in the Relative Differences between Tables 6 and 7 are related to the use of the measured results to extract the equations (Table 7) instead of equations based on the modelling stages (Table 6). This means that the differences between errors presented in Table 7 and those exhibited in Table 6 are related to the modelling stages: solutions characterization and their mathematical modelling on one hand and sensor numerical simulations using these material models on the other hand.

Moreover, the resulting errors presented in Table 7 could be implied by either experimental inaccuracies or related to the regression coefficients during the equation extraction step.

To evaluate the contribution of experimental inaccuracies upon the resulting error, several measurements of some aqueous solution samples were made in a row, all external parameters being kept identical. The resulting relative errors between values extracted from two consecutive measurements appeared to reach up to 3.9%, yielding quadratic differences up to 5.4%. These results corroborate the hypothesis of experimental electrical measurement inaccuracies, which could be attributed to, amongst others, the measurement equipment or the rinsing procedures.

Additionally, errors could also come from the samples themselves, their exact composition and concentrations being unknown. Indeed, as described in “Dielectric characterization and modelling of aqueous solutions” section, samples were prepared by dissolving the ad-hoc weighted compounds under powder form into DI-water. This process might introduce errors, which would be consistent with the fact that errors appear to be consistently on the same samples.

## Conclusions

Microwave characterization and modelling of binary solutions involving sodium chloride and water on one hand and sucrose and water on the other hand have been presented, occurring physical phenomena being described. Then, ternary mixtures made of sucrose, sodium chloride and water at various concentrations have been electromagnetically characterized over a wide frequency band, yielding an EM model parametrized by the sucrose and sodium chloride concentrations.

Based upon these models, a bi-parameter microwave sensor able to track both sucrose and sodium chloride in aqueous solutions has been introduced. Its design relies on two electrically-coupled open-loop (SRR) resonators, the sample being located in the inter-coupling area. The sensor design parameters have been optimized during a retrofitting stage to finely adjust the 3D model of the sensor with measurements. This calibration stage was made using air and DI-water as references. Afterwards, sensing parameters have been discussed and chosen, they consist in the reflected S-parameter and a Sensing Ratio relying on the central frequency, bandwidth and transmitted S-parameter, which is calculated around 7 GHz. Then, full-wave simulations of the sensor were run with the previously-described models for samples exhibiting various concentrations of sucrose and sodium chloride. From the simulation results, predictive variation laws of the sensing parameters with respect to the compounds concentrations have been set. Finally, real samples have been measured and the predicted concentrations values have been compared to the real ones, demonstrating the ability of the sensor to retrieve concentrations with a rather good accuracy within the measured ranges.

Further work could include enhancement of the sensitivity through the improvement of the sensor design to address lower concentration ranges, which could be more suitable for potential industrial applications. Finally, the design of a microwave sensor able to monitor more than two compound concentrations is also a worthwhile challenge by considering the craving demand of highly sensitive, continuously-measuring sensors.
